# Prognostic Value and Staging Classification of Retropharyngeal Lymph Node Metastasis in Nasopharyngeal Carcinoma Patients Treated with Intensity-modulated Radiotherapy

**DOI:** 10.1371/journal.pone.0108375

**Published:** 2014-10-10

**Authors:** Ling-Long Tang, Rui Guo, Guanqun Zhou, Ying Sun, Li-Zhi Liu, Ai-Hua Lin, Haiqiang Mai, Jianyong Shao, Li Li, Jun Ma

**Affiliations:** 1 Department of Radiation oncology, Sun Yat-sen University Cancer Center; State Key Laboratory of Oncology in South China; Collaborative Innovation Center for Cancer Medicine, Guangzhou, Guangdong, People's Republic of China; 2 Imaging Diagnosis and Interventional Center, State Key Laboratory of Oncology in South China, Sun Yat-sen University Cancer Center, Collaborative Innovation Center for Cancer Medicine, Guangzhou, Guangdong, People's Republic of China; 3 Department of Medical Statistics and Epidemiology, School of Public Health, Sun Yat-sen University, Guangzhou, Guangdong, People's Republic of China; 4 Department of Nasopharyngeal Carcinoma, Sun Yat-Sen University Cancer Center, Collaborative Innovation Center for Cancer Medicine, Guangzhou, Guangdong, People's Republic of China; 5 Department of Molecular Diagnostics, State Key Laboratory of Oncology in South China, Sun Yat-Sen University Cancer Center, Collaborative Innovation Center for Cancer Medicine, Guangzhou, Guangdong, People's Republic of China; National Cancer Center, Japan

## Abstract

**Background:**

The development of intensity-modulated radiotherapy (IMRT) has revolutionized the management of nasopharyngeal carcinoma (NPC). The purpose of this study was to evaluate the prognostic value and classification of TNM stage system for retropharyngeal lymph node (RLN) metastasis in NPC in the IMRT era.

**Material and Methods:**

We retrospectively reviewed data from 749 patients with biopsy-proven, non-metastatic NPC. All patients received IMRT as the primary treatment. Chemotherapy was administered to 86.2% (424/492) of the patients with stage III or IV disease.

**Results:**

The incidence of RLN metastasis was 64.2% (481/749). Significant differences were observed in the 5-year disease-free survival (DFS; 70.6% vs. 85.4%, *P*<0.001) and distant metastasis-free survival (DMFS; 79.2% vs. 90.1%, *P*<0.001) rates of patients with and without RLN metastasis. In multivariate analysis, RLN metastasis was an independent prognostic factor for disease failure and distant failure (*P* = 0.005 and *P* = 0.026, respectively), but not for locoregional recurrence. Necrotic RLN metastases have a negative effect on disease failure, distant failure and locoregional recurrence in NPC with RLN metastasis (*P* = 0.003, *P* = 0.018 and *P* = 0.005, respectively). Survival curves demonstrated a significant difference in DFS between patients with N0 disease and N1 disease with only RLN metastasis (*P* = 0.020), and marginally statistically significant differences in DMFS and DFS between N1 disease with only RLN metastasis and other N1 disease (*P* = 0.058 and *P* = 0.091, respectively). In N1 disease, no significant differences in DFS were observed between unilateral and bilateral RLN metastasis (*P* = 0.994).

**Conclusions:**

In the IMRT era, RLN metastasis remains an independent prognostic factor for DFS and DMFS in NPC. It is still reasonable for RLN metastasis to be classified in the N1 disease, regardless of laterality. However, there is a need to investigate the feasibility of classifying RLN metastasis as N1a disease in future by a larger cohort study.

## Introduction

Although nasopharyngeal carcinoma (NPC) is rare in most regions of the world, it is endemic in certain regions, especially Southeast Asia. The incidence of NPC is approximately 30–80 per 100,000 per year in Southern China [1]. The nasopharynx contains a well-developed network of lymph nodes, and the retropharyngeal lymph nodes (RLN) are regarded as one of the key lymph nodes in NPC [2]. Due to the limitations of CT imaging, RLN metastases are isodense and contiguous with the primary tumor, and can be difficult to identify as a separate mass on CT scans; therefore, the true incidence of RLN metastasis was previously underestimated [3]–[5]. However, due the superior soft tissue contrast and multiplanar capacity of MRI, the incidence of RLN metastasis in NPC is currently approximately 70% [5], [6], and RLN metastasis has received increasing attention in recent years.

The recent seventh edition of the International Union against Cancer/American Joint Committee on Cancer (UICC/AJCC) staging system for NPC incorporated RLN metastasis into the tumor-node-metastasis (TNM) classification, and classified it as N1 disease regardless of laterality [7], [8]. However, this revision was based on patients treated with a two-dimensional conventional radiotherapy technique. Intensity-modulated radiotherapy (IMRT) offers an improved tumor target conformity, allows safer dose escalations and yields superior results in NPC compared to 2D-CRT, primarily by achieving a higher local tumor control rate [9]. IMRT has gradually replaced two-dimensional conventional radiotherapy as the primary radiotherapy modality for the treatment of NPC; however, the introduction of new therapeutic technologies may require a revaluation of the prognostic value and appropriate classification of TNM staging system for RLN metastasis in NPC.

In this study, we analyzed the outcomes of patients with NPC staged by MRI prior to treatment who subsequently received IMRT and the current standard systemic treatments, to investigate whether it is still reasonable to classify RLN metastasis in NPC as N1 disease in the IMRT era.

## Methods and Materials

### Patient characteristics

Approval for retrospective analysis of the patient data was obtained from the ethics committee of Sun Yat-sen University Cancer Center. Written consent was waived, while oral consent from the patients was obtained via telephone and documented by telephone recording. All 749 patients with newly diagnosed, biopsy-proven, non-metastatic NPC who were treated at Sun Yat-Sen University Cancer Center using IMRT between January 2003 and December 2007 were retrospectively reviewed. The clinicopathologic characteristics of the patients are shown in Table 1.

**Table 1:** The clinicpathological characters of 749 patients in this study.

Characteristic	N	Column (%)
Age		
>50	553	73.8
≤50	196	26.2
Gender		
Male	580	77.4
Female	169	22.6
Histologic type		
WHO II/III	744	99.3
WHO I	5	0.7
T category		
T1	177	23.6
T2	140	18.7
T3	264	35.2
T4	168	22.4
N category		
N0	184	24.6
N1	409	54.6
N2	106	14.2
N3	50	6.7
Stage		
I	78	10.4
II	179	23.9
III	282	37.7
Iva-b	210	28.0
Chemotherapy		
No	214	28.6
Concurrent	233	33.5
Concurrent + Induction	246	32.8
Concurrent + Adjuvant	46	6.1
PET-CT		
Yes	162	21.6
No	587	78.4

All patients completed a pre-treatment evaluation including complete patient history, physical examination, hematology and biochemistry profiles, neck and nasopharyngeal MRI, chest radiography, abdominal sonography and a single photon emission computed tomography (SPECT) whole body bone scan. 18-F-fluorodeoxyglucose (FDG)-positron emission tomography (PET)-CT was performed on 162/749 patients (21.6%). All patients were retrospectively re-staged according to the 7th edition of UICC/AJCC staging system. The distribution of disease stages was: stage I, 10.4%; stage II, 23.9%; stage III, 37.7% and stage IVa–b, 28.0% (Table 1).

### MRI techniques and criteria for retropharyngeal lymph node and other cervical lymph node metastasis

All patients underwent MRI scans using a 1.5-T system (Signa, General Electric, CV/i; General Electric Healthcare, Chalfont St. Giles, United Kingdom). The area from the suprasellar cistern to the inferior margin of the sternal end of clavicle was examined with a head and neck combined coil. T1-weighted fast spin-echo images in the axial, coronal and sagittal planes (repetition time of 500–600 ms and echo time of 10–20 ms), and T2-weighted fast spin-echo MR images in the axial plane (repetition time of 4000–6000 ms and echo time of 95–110 ms) were obtained before injection of contrast material. After intravenous injection of Gd-DTPA at a dose of 0.1 mmol/kg body weight, spin-echo T1-weighted axial and sagittal sequences, and spin-echo T1-weighted fat-suppressed coronal sequences were performed sequentially using parameters similar to those used before injection of contrast. The section thickness for the axial plane was 5 mm with a 1 mm interslice gap, and 6 mm with a 1 mm interslice gap for the coronal and sagittal planes.

Two radiologists specializing in head and neck cancers evaluated all of the scans independently. Any disagreements were resolved by consensus. The diagnostic MRI criteria for metastatic lymphadenopathy included: 1) lateral RLN with a minimal axial diameter of ≥5 mm and any node seen in the median retropharyngeal group, or lymph nodes with a minimal axial diameter ≥11 mm in the diagastric region or ≥10 mm for all other cervical nodes except the retropharyngeal group; 2) lymph nodes of any size with central necrosis or a contrast-enhanced rim; and 3) nodal grouping: the presence of three or more contiguous and confluent lymph nodes, each of which should have a minimal axial diameter of 8–10 mm [10]–[12]. The criteria for extranodal neoplastic spread (ENS) in RLN was the presence of indistinct nodal margins, irregular nodal capsular enhancement or infiltration into the adjacent fat or muscle [13].

### Treatment

All patients received IMRT as the primary treatment. The patients were immobilized in supine position by a thermoplastic mask. After administration of intravenous contrast material, 3-mm CT slices, depicting the area of the head until 2 cm below the sterno-clavicular joint, were acquired. The primary tumor and the upper-neck area above the caudal edge of the cricoid cartilage were treated by IMRT. Target volumes were in agreement with the International Commission on Radiation Units and Measurements Reports 50 and 62. The contoured images were transferred to an integrated IMRT planning and delivery system (Peacock, Corvus 3.0, NOMOS Corporation, Sewickley, Pa). The prescribed radiation dose was defined as follows: a total dose of 68 Gy in 30 fractions at 2.27 Gy per fraction to the planning target volume (PTV) of the primary gross tumor volume (GTV-P), 60 to 64 Gy to the PTV of nodal gross tumor volume (GTV-N), 60 Gy to the PTV of CTV-1 (i.e., high-risk regions), and 54 Gy to the PTV of CTV-2 (i.e., low-risk regions) and CTV-N (i.e., neck nodal regions). The treatment was delivered by a dynamic, multileaf, intensitymodulating collimator (called MIMiC). For the lower neck, an anterior cervical field was used. All patients were treated with one fraction daily over 5 days per week.

Chemotherapy was administered to 86.2% (424/492) of the patients with stage III or IV disease. The chemotherapy regimens included concurrent chemotherapy alone, concurrent chemotherapy combined with induction chemotherapy and/or adjuvant chemotherapy in conjunction with a platinum-based therapeutic clinical trial. Reasons for deviation from institutional guidelines included patients' refusal, age (≥70 years), organ severe dysfunction (diabetes, cardiac dysfunction, renal insufficiency, liver insufficiency, et al) that would suggest intolerance to chemotherapy. When possible, salvage treatments such as intracavitary brachytherapy, surgery and chemotherapy were provided in the event of documented relapse or persistent disease.

### Follow-up

Median follow-up was 81 months (range, 3–127 months). Each patient was assessed weekly during treatment for treatment response and toxicity, and every 2–3 months during the first 2 years and every 3–6 months during years 3–5 after radiotherapy. Endoscopy, CT or MRI scans of the head and neck were performed every 3 months during the first year and annually during years 2–5. Patients with residual or recurrent local disease underwent biopsy to confirm malignancy. Additional tests were ordered when indicated to evaluate for local or distant failure.

### Statistical analysis

All analyses were performed using SPSS version 20.0 (IBM Corporation, Armonk, NY, USA). Actuarial rates were estimated by the Kaplan-Meier method; survival curves were compared using the log-rank test [14]. The following endpoints (measured from the start of treatment to the first defining event) were estimated: locoregional relapse-free survival (LRRFS), local relapse-free survival (LRFS), and nodal relapse-free survival (NRFS), DMFS, disease-free survival (DFS) and overall survival (OS).

Multivariate analyses with the Cox proportional hazards model were used to test for independent significance by backward elimination of insignificant explanatory variables [15]. The Cox proportional hazards model was used to calculate hazard ratios (HRs). Two-tailed *P* values <0.05 were considered statistically significant.

## Results

### Incidence of RLN metastasis

In this study, no patient had a metastatic median RLN, and the incidence of lateral RLN metastasis in the current study was 64.2% (481/749 patients). Thirty-two percent (154/481) of patients with RLN metastasis had no evidence of cervical lymph node (CLN) metastasis, and 79.6% (327/411) patients with CLN metastasis had evidence of RLN involvement.

Of 481 the patients with RLN metastasis, 63.2% (304/481) had unilateral RLN involvement, whereas 36.8% (177 of 481) had bilateral involvement. The mean minimal and maximal axial diameters of the RLN metastases were 9.61±4.31 mm (range, 5–28 mm) and 12.66±5.61 mm (range, 5–36 mm). The incidence of RLN necrosis was 13.3% (64/481) and the incidence of ENS was 21.8% (105/481).

### Prognostic value of RLN metastasis

There were 56/749 (7.5%) patients developed recurrence, including 34 patients (4.5%) with isolated local recurrences, 15 patients (2.0%) with isolated regional nodal recurrences, and 7 patients (0.9%) with both local and regional nodal recurrence. In addition, there were 129 (17.2%) patients developed distant metastases and 149 (19.9%) died. The 5-year survival rates were: LRRFS, 92.9%; DMFS, 83.1%; DFS, 75.9% and OS, 83.9%.

**Figure 1:**
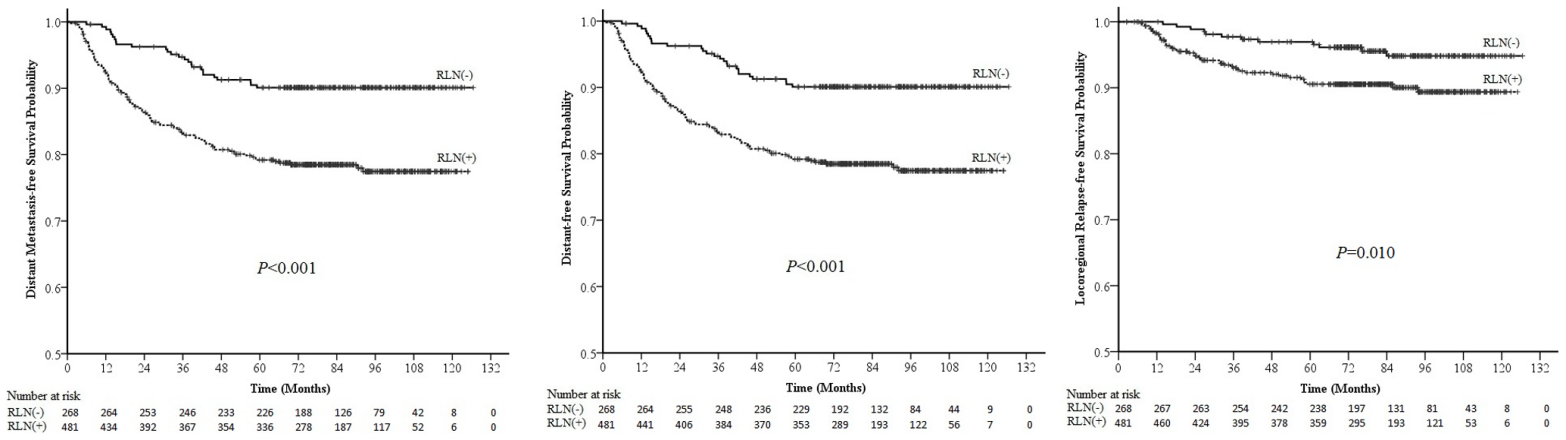
Survival curves for nasopharyngeal carcinoma (NPC) patients with and without retropharyngeal lymph node (RLN) metastasis. RLN (-): NPC patients without RLN metastasis; RLN (+): NPC patients with RLN metastasis.

Significant differences were observed in the 5-year DFS (70.6% vs. 85.4%, *P*<0.001), DMFS (79.2% vs. 90.1%, *P*<0.001) and LRRFS (90.5% vs. 97.0%, *P* = 0.010) rates of patients with and without RLN metastasis (Figure 1). Multivariate analysis was performed to adjust for various prognostic factors; the following known important prognostic variables were included in the Cox proportional hazards model: age (≤50 vs.>50 years), gender, T-classification, chemotherapy (yes vs. no), bilateral CLN metastasis (yes vs. no), dimension of CLN metastases (≤6 vs.>6 cm) and the location of CLN metastasis (with supraclavicular lymph nodes metastasis vs. without supraclavicular lymph nodes metastasis). Dimension of CLN metastases (≤6 vs.>6 cm) was measured based on maximal diameter by palpation. RLN metastasis was an independent prognostic factor for disease failure and distant failure (HR = 1.663, 95% CI: 1.169–2.365, *P* = 0.005, and HR = 1.682, 95% CI: 1.065–2.655, *P* = 0.026, respectively), but not for locoregional recurrence (Table 2).

**Table 2:** Summary of multivariate analysis of prognostic factors in 749 patients with nasopharyngeal carcinoma.

Endpoint	Variable	B	*P* *	HR	95% *CI* for HR
Distant failure	Retropharyngeal lymph node (yes vs. no)	0.520	0.026	1.682	1.065–2.655
	Age (≤50 vs. 50 years)	0.406	0.040	1.501	1.019–2.211
	T-classification	0.451	<0.001	1.570	1.307–1.887
	CLN dimension (≤6 vs. > 6 cm)	1.268	<0.001	3.555	1.965–6.434
	CLN location (with SCLN vs. without SCLN)	0.896	0.003	2.450	1.351–4.443
	Bilateral CLN (yes vs. no)	0.511	0.013	1.668	1.114–2.497
Disease failure	Retropharyngeal lymph node (yes vs. no)	0.509	0.005	1.663	1.169–2.365
	Age (≤50 vs. 50 years)	0.656	<0.001	1.927	1.427–2.602
	T classification	0.435	<0.001	1.544	1.335–1.787
	CLN dimension (≤6 vs. >6 cm)	1.068	<0.001	2.908	1.691–5.001
	CLN location (with SCLN vs. without SCLN)	0.818	0.002	2.266	1.342–3.826
	Bilateral CLN (yes vs. no)	0.362	0.040	1.436	1.016–2.029
Locoregional recurrence	Retropharyngeal lymph node (yes vs. no)	*NS*			
	T-classification	0.438	0.001	1.550	1.183–2.031
	Bilateral CLN (yes vs. no)	0.681	0.022	1.975	1.105–3.530

Abbreviations: CI  =  confidence interval; HR =  hazard ratio; CLN  =  cervical lymph nodes; NS  =  not significant; SCLN = Supraclavicular lymph node.

*P values were calculated using an adjusted Cox proportional-hazards model. The following known important prognostic variables were included in the Cox proportional hazards model: age (≤50 vs. >50 years), gender, T-classification, chemotherapy (yes vs. no), bilateral CLN metastasis (yes vs. no), dimension of CLN metastases (≤6 vs. >6 cm), CLN location (with SCLN vs. without SCLN) and RLN metastasis (yes vs. no).

All of the MRI-determined nodal variables were analyzed in the 481 patients with RLN metastasis using univariate analyses and multivariate analyses. The RLN variables were categorized as follows: minimal axial diameters (<10 vs. ≥10 mm MID), necrosis (no vs. yes), laterality (unilateral vs. bilateral) and ENS (no vs. yes). Univariate analysis revealed that necrosis had significant prognostic value for DMFS, DFS and LRRFS (*P*<0.001, *P*<0.001 and *P*<0.001; Table 3). After adjusting for various prognostic factors including age, sex, T-classification, N-classification and chemotherapy, necrosis remained significant for disease failure, distant failure and locoregional recurrence (HR = 1.795, 95%CI: 1.214–2.654, *P* = 0.003; HR = 1.752, 95%CI:1.100–2.790, *P* = 0.018 and HR = 2.614, 95%CI: 1.339–5.103, *P* = 0.005; Table 4).

**Table 3:** Five-year survival rates for 481 nasopharyngeal carcinoma patients with retropharyngeal lymph nodes metastasis according to the characteristics of retropharyngeal lymph node metastasis.

	Size (minimal axial diameters)			Necrosis			Laterality			Extranodal neoplastic spread		
	<10 mm	≥10 mm	*P* *	No	Yes	*P* *	Unilateral	Bilateral	*P* *	No	Yes	*P* *
DMFS	86.5	71.9	<0.001	81.6	62.8	<0.001	82.5	73.6	0.032	81.6	70.3	0.001
DFS	78.3	62.9	<0.001	73.3	53.1	<0.001	73.6	65.5	0.054	74.1	58.1	0.002
LRRFS	93.5	87.3	0.017	92.0	80.0	<0.001	91.5	88.9	0.310	92.0	85.2	0.019

Abbreviations: DFS  =  disease-free survival; DMFS  =  Distant metastasis-free survival; LRRFS  =  Locoregional relapse-free survival.

*P values were calculated by the unadjusted log-rank test.

**Table 4:** Summary of multivariate analysis of prognostic factors in 481 nasopharyngeal carcinoma patients with retropharyngeal lymph node metastasis (RLN) metastasis.

Endpoint	Variable	B	*P* *	HR	95% CI for HR
Disease failure	Age (≤50 vs. 50)	0.659	<0.001	1.933	1.374–2.721
	T-classification	0.330	<0.001	1.391	1.167–1.658
	N-classification	0.349	<0.001	1.417	1.203–1.670
	Necrosis	0.585	0.003	1.795	1.214–2.654
	MID (<10 vs. ≥10 mm)	0.329	0.063	1.389	0.983–1.964
Distant failure	Age (≤50 vs. 50 years)	0.412	0.064	1.510	0.976–2.335
	T-classification	0.318	0.004	1.374	1.107–1.706
	N-classification	0.413	<0.001	1.511	1.249–1.828
	Necrosis	0.561	0.018	1.752	1.100–2.790
	MID (<10 vs. ≥10 mm)	0.490	0.026	1.632	1.059–2.515
Locoregional recurrence	Necrosis	0.961	0.005	2.614	1.339–5.103
	MID (<10 vs. ≥10 mm)	0.570	0.083	1.767	0.929–3.364

Abbreviations: CI  =  confidence interval, MID = minimal axial diameters.

*P values were calculated using an adjusted Cox proportional-hazards model. The following known important prognostic variables were included in the Cox proportional hazards model: minimal axial diameters of RLN (<10 vs. ≥10 mm MID), necrosis of RLN (no vs. yes), laterality of RLN (unilateral vs. bilateral) and extra nodal neoplastic spread of RLN (no vs. yes), age (≤50 vs. 50), sex, T-classification, N-classification and chemotherapy (no vs. yes).

### Survival according to N classification

**Figure 2:**
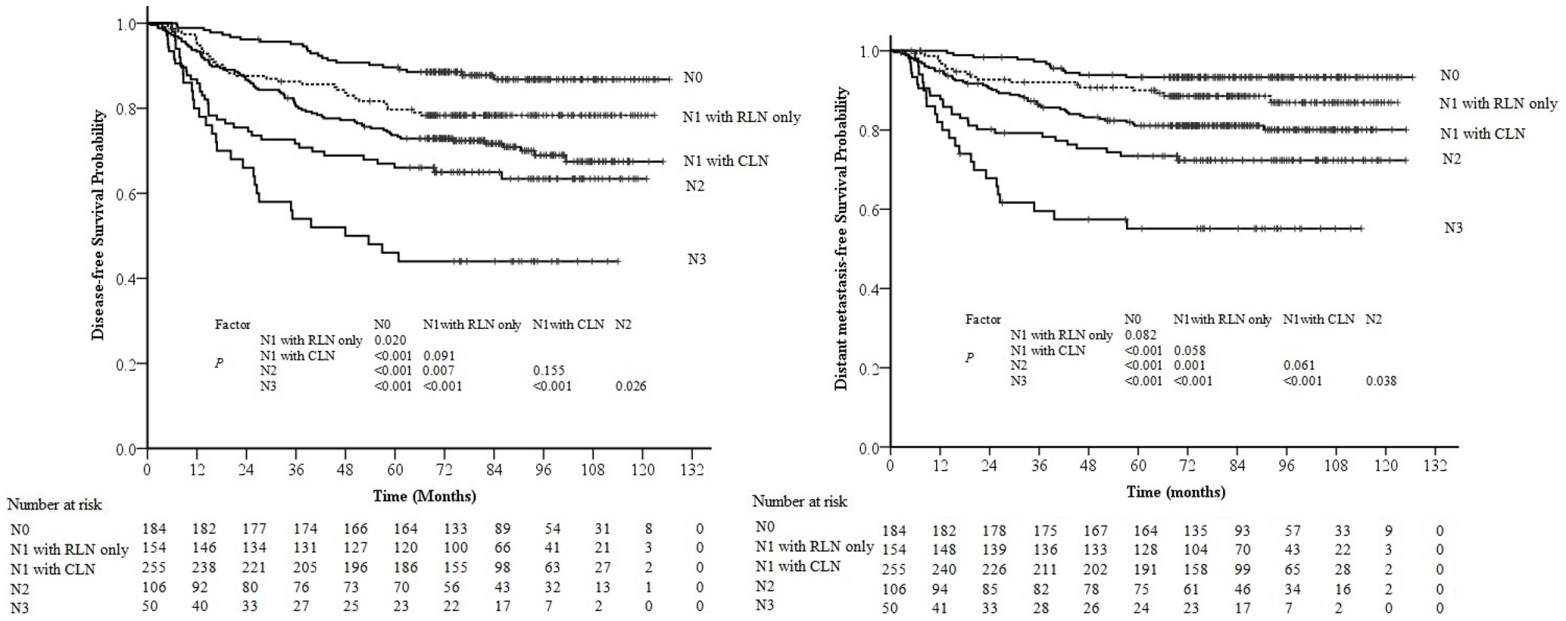
Survival curves for patients with nasopharyngeal carcinoma (NPC) stratified by the N classification of the 7^th^ edition of the UICC/AJCC staging system for NPC. N1 + RLN only: N1 disease with retropharyngeal lymph node metastasis and without cervical lymph node metastasis; N1 + CLN: N1 disease with cervical lymph node metastasis.

According to the seventh edition of AJCC staging system, RLN is included as a criterion for N1 disease, and 154 (20.6%) N0 patients would be upgraded to N1 disease (N1 with RLN only). All 749 patients were divided into five groups: N0 disease, N1 disease with retropharyngeal lymph node metastasis and without CLN metastasis (N1 with RLN only), N1 disease with CLN metastasis (N1 with CLN), N2 disease, and N3 disease. The survival curves demonstrated a significant difference in DFS between patients with N0 disease and N1 with RLN only (*P* = 0.020). The differences in DMFS and DFS between N1 with RLN only and N1 with CLN were marginally statistically significant (*P* = 0.058 and *P* = 0.091, respectively; Fig. 2).

In the N1 disease group, no significant differences were observed in the DFS, MDFS, LRFS or NRFS rates of patients with unilateral and bilateral RLN metastasis (*P* = 0.994, *P* = 0.752, *P* = 0.398 and *P* = 0.08 respectively).

## Discussion

The TNM staging system is crucial for predicting prognosis, guiding treatment strategy for different risk groups, and facilitating the exchange of information between oncology centers [8], [16]. The TNM staging system is continually being modified to account for new developments in diagnostic and therapeutic techniques. There is little controversy that IMRT is the treatment of choice for NPC, as dosimetric studies have demonstrated the clear advantages of IMRT in terms of improving the dose conformity for complex tumor targets and better protecting the adjacent organs at risk [17], [18]. This is the first study to demonstrate that it is reasonable for RLN metastasis in NPC to be classified as N1 disease in the IMRT era.

### Prognostic value of RLN metastasis

Several studies have reported that neck lymph node involvement in NPC spreads in an orderly manner down the neck [19], [20]. RLN metastasis is very common in NPC, as the RLNs are the first echelon lymph node [21]. When treated with two-dimensional conventional radiotherapy, patients with NPC and RLN metastasis had a poor prognosis [7]. In this study of patients treated with IMRT, RLN metastasis remained an independent prognostic factor for DFS and DMFS, even after adjustment for various prognostic factors. It is possible that conventional two-dimensional and three-dimensional conformal radiotherapy and IMRT do not have a significantly different effect on DMFS in patients with RLN metastasis. A number of studies have confirmed that IMRT has improved local control, but not distant control, in NPC [9], [17], [22]. In this study, the DMFS rate was only 83.1%, indicating that distant failure remains a challenge in patients with RLN metastasis. Therefore, the inclusion of RLN metastasis in the UICC/AJCC staging system would be useful to guide treatment planning, and additional therapeutic improvements are required to achieve a favorable outcome in patients with RLN metastasis.

Central necrosis is considered to be a late event in the biological evolution of tumor metastases within lymph nodes [23]. It has been confirmed that central necrosis primarily occurs in lymph nodes approximately 20.0 mm or larger, and it appears that central necrosis characteristically occurs after massive tumor infiltration [24]. There are few reports about the prognostic value of necrosis in RLN metastases in NPC. In this study, we found that necrosis of RLN metastases had a negative effect on survival in NPC. Tumor necrosis is believed to represent the endpoint of severe, chronic hypoxia in tissues distal to functional blood vessels. Tumor hypoxia may be one factor accounting for the poor prognosis of patients with necrotic RLN metastases [25]. Although IMRT offers improved tumor target coverage, the lack of oxygen in areas of hypoxia not only makes the tissues less susceptible to radiotherapy, but also induces the transcription of a variety of genes which promote tumor progression and increase tumor aggressiveness compared to non-hypoxic tumors [26], [27], which may explain why necrosis had significant prognostic value for all endpoints (DFS, DMFS and LRRFS). We propose that RLN necrosis should be adopted as a factor to enhance individualized NPC patient prognostication and clinical decision making, especially as it is simple to assess and could easily be incorporated into routine histopathological examinations.

### Classification for RLN metastasis

Due to the limited diagnostic capabilities prior to the era of MRI imaging, consistent guidelines for the designation of RLN metastasis could not be identified in previous TNM staging systems; RLN metastasis was only incorporated into the TNM classification of the most recent 7th edition of the UICC/AJCC staging system for NPC. Evidence from two retrospective studies indicated that patients with RLN alone, regardless of laterality, have a similar risk of distant metastasis (DM) as patients with N1 disease [7], [28]. However, all of the patients in one of these studies [7] underwent conventional radiotherapy, and in the other study [28], only 12.7% of the patients underwent IMRT and there were no stratification analyses according to the radiation technique. IMRT achieves a significantly higher survival rate in NPC than CRT [9]; therefore, it was necessary to reevaluate whether it is still reasonable for RLN metastasis to be classified as N1 disease in the IMRT era.

This study, in which all patients received IMRT as the primary treatment, demonstrates that it is still reasonable for RLN metastasis in NPC to be classified as N1 disease, regardless of laterality. There are a number of reasons for this suggestion: Firstly, it is well recognized that the RLNs are the first echelon node in NPC. In most cases, and unlike CT, MRI can discriminate the RLNs from the primary tumor, so RLN metastasis should be classified in the N-classification, not the T-classification. Secondly, the survival curves in this study demonstrated a significant difference in DFS between patients with N0 disease and N1 disease with RLN only; therefore, it is still reasonable for RLN metastasis to be classified as N1 disease in patients treated with IMRT. Lastly, no significant differences were observed in the DFS, MDFS and LRFS rates of patients with unilateral and bilateral RLN metastasis, so RLN laterality does not need to be considered in future revisions of the staging system.

However, it was also observed that the differences in DMFS and DFS between N1 with RLN only and N1 with CLN were marginally statistically significant, and the survival curve could be separated, so there is a need to investigate the feasibility of classifying RLN metastasis as N1a disease in future by a larger cohort study.

It should be emphasized that this study was retrospective, and these results should be confirmed in a prospective study in the future. However, the current analysis of a large number of patients who received a systemic staging workup and were treated at single institution offers valuable information for evaluating appropriate classification of TNM stage for RLN metastasis in the current MRI/IMRT era. More importantly, all patients were treated with IMRT, in compliance with the requirements for studies aimed at formulating improvements in staging systems [16].

In conclusion, in the IMRT era, RLN metastasis remains an independent prognostic factor in NPC, and necrotic RLN metastases have a negative effect on survival in NPC. It is still reasonable for RLN metastasis to be classified in the N1 disease, regardless of laterality. However, there is a need to investigate the feasibility of classifying RLN metastasis as N1a disease in future by a larger cohort study.
